# Susceptibility of Cider Apple Accessions to European Canker—Comparison between Evaluations in Field Planted Trees and Rapid Screening Tests

**DOI:** 10.3390/plants11091145

**Published:** 2022-04-23

**Authors:** Álvaro Delgado, Belén García-Fernández, Antonio Gómez-Cortecero, Enrique Dapena

**Affiliations:** 1 Servicio Regional de Investigación y Desarrollo Agroalimentario (SERIDA), Apdo.13, E-33300 Villaviciosa, Spain; bgarcia@serida.org (B.G.-F.); edapena@serida.org (E.D.); 2 NIAB, Lawrence Weaver Rd., Cambridge CB3 0LE, UK; antonio.gomez@niab.com

**Keywords:** *Neonectria ditissima*, field assessment, artificial inoculations, local cultivars, germplasm repository

## Abstract

European canker, caused by *Neonectria ditissima* Bres., is an economically damaging fungal disease of apple. Breeding new cultivars with a high level of resistance to European canker is the main aim of apple breeding programs. Observations of symptoms in naturally infected trees were carried out in 400 apple accessions in Asturias (north-western Spain). Young and mature field planted trees were assessed under conditions highly conducive for *N. ditissima* development. The results demonstrated that juvenile trees (4-year-old) barely showed noticeable symptoms whereas a wide variability in the levels of resistance among accession was observed in mature trees (14-year-old). Around 28% of the locally maintained collection resulted to be highly resistant to this disease in the region. Field observations on mature trees were also compared to four rapid screening tests based on artificially induced lesions. Spearman correlation analysis using two resistance parameters revealed that none of the methods resulted in similar rankings of cultivar susceptibility as some accessions that were ranked as resistant for a given test turned out to be susceptible in the field. This study might suggest that whilst conventional resistance phenotyping techniques are time-consuming, the outcomes of this approach still seem the preferred option to assess the response to *N. ditissima* of apple accessions.

## 1. Introduction

European canker caused by the phytopathogenic fungus *Neonectria ditissima* Bres. (formerly known as *Nectria galligena*) affects several tree fruit hosts including apple (*Malus domestica* Borkh.) and causes a substantial economic damage worldwide in pome fruit crops. The formation of cankers on woody tissues is the most obvious symptom. Cankers can girdle the main stem and this fungus is also able to cause the death of buds, shoots, spurs, and branches [1,2,3,4]. A combination of symptoms can deteriorate the tree architecture and branching habit, which eventually might lead to the death of part of the tree or even the whole tree [5]. In any case, the consequences often result in a reduction of tree vigour and fruit yield [4]. Some authors also reported that *N. ditissima* causes fruit rotting, either in the orchard or later during storage [6,7] and, in some settings, cankered wounds can be a shelter of pests. *N. ditissima* can reproduce sexually or asexually depending on the environmental conditions [8,9]. Conidia (i.e., asexual spores) are washed and dispersed by rain splash from first year canker formations during autumn, when they generally infect the plants [10]. Ascospores (i.e., sexual spores) from older canker lesions are released and dispersed by wind or rain during rainy episode in spring and summer [11]. Wounds are the main entry point for this pathogen and wounds can be either natural, such as bud-scale scars, leaf scars, and fruit scars, or artificial, as in the case of pruning wounds.

Rain and moisture are the biggest limiting factors for *N. ditissima* as they are essential for spore production, germination, and infection [7]. Several authors reported that the duration of rainfall and surface wetness are more important than amount of rainfall [12,13,14,15]. Since inoculum and points of entry on the tree are available during all growth stages [16] an effective control of apple canker remains difficult [3,17]. Control of *N. ditissima* relies primarily on pruning out the cankered lesions and fungicide sprays. Given that the goal of sustainable fruit production is to reduce the negative effects of pesticides [18], the selection of resistant or low susceptible genotypes is a promising long-term alternative for growing apple trees with lower inputs of pesticides. Considering that a total resistance to canker has not yet been reported among apple cultivars or wild *Malus* relatives [5,19], the identification of genotypes with an optimal level of resistance to this disease has become increasingly important to apple breeders.

Asturias is a region in north-western Spain with a long tradition in apple growing and cider-making. The Apple Germplasm Bank of Asturias maintained by SERIDA (Servicio Regional de Investigación y Desarrollo Agroalimentario), currently maintains 550 local apple accessions [20]. The region has a humid temperate Atlantic climate and forms part of the “Green Spain” where high precipitation occurs all year round, summers are mild, and winters also have relatively benign temperatures, rarely including frost in the apple growing regions [21]. The highest levels of rain are recorded between November and February [22], but there is frequent rainfall at any time of the year, with an average of 128 days per year with a daily precipitation above 1 mm (data retrieved from the meteorological station of the Asturias airport managed by AEMET). As a result of these climatic characteristics, the conditions for infection by *N*. *ditissima* are especially favourable in the region. On the other hand, organic apple production has been gaining popularity over the recent years (COPAE, personal communication) and conventional orchards are also managed under low pesticide-input cropping systems. For these reasons, one of the main aims of the SERIDA Fruit Research Program is to obtain tolerant cultivars that are suitable for organic production and can make the use of fungicides unnecessary or infrequent.

A wide genetic variation in the levels of susceptibility to European canker has been documented among apple cultivars and wild *Malus* species [19,23,24,25,26,27,28]. The evaluation of the susceptibility to European canker based on phenotyping natural infections in the field have been previously conducted in fruit research institutes [29,30,31,32]. However, the results are largely influenced by local climatic conditions, especially rainfall and temperature during the infection periods [12]. The age of wounds, amount, and source of fungus inoculum and orchard management practices including pruning and fertilisation are other factors that play a significant role in the severity of the disease [4]. As a result, cultivars can be described as resistant or susceptible depending on the environmental and experimental conditions [33]. Additionally, this approach is a long-term process that implies waiting several years until the trees show enough symptoms to make an appropriate decision in terms of its field susceptibility. At SERIDA, the differences in the relative levels of resistance are determined by measuring natural infections on mature trees whose age ranges between preferably 9 and 10-year-old [26].

Given that the implementation of molecular markers for the selection of resistance genes for *N. ditissima* is not yet a viable approach, adequate artificial methods for a rapid selection of genotypes under controlled conditions are expected to accelerate and make the breeding process more efficient, resulting in an easier and cheaper evaluation of cultivar susceptibility. Studies on detached shoots and potted trees as fast-phenotyping methods are currently a standard practice [19,25,27,28,34], but the accuracy in the quantification of the levels of resistance is not entirely clear and results often show inconsistent resistance classifications compared to results obtained by phenotyping adult trees [19,34]. The aims of this study were (1) to evaluate the different levels of susceptibility to *N. ditissima* among 400 local apple accessions under natural field conditions; (2) to test the pathogenicity of 3 isolates collected in different locations of Asturias; (3) to evaluate the reliability of a range of rapid screening tests and resistance parameters and (4) to identify genotypes that can be used as progenitors in breeding programs due to their resistance to European canker.

## 2. Results

### 2.1. Field Phenotyping in Young and Mature Apple Trees

Our visual observations in mature apple trees confirm the importance of natural infections of *N. ditissima* in north-western Spain. All studied accessions were injured by European canker, but the level of damage varied greatly among genotypes. The analysis of the disease severity scores based on an ordinal scale estimating the severity of the disease on mature trees (14-year-old) revealed a wide variability in terms of the susceptibility to *N. ditissima* (Table 1). Nearly 97% of the collection showed symptoms and the mean level across the 400 accessions was 2.21 ± 0.91. A low mean coefficient of variation (17.8%) indicates that the variability between individuals with the same genotype was small. When the same procedure was carried out in young field planted trees (4-year-old), only 15 accessions exhibited symptoms, which represents around 3.8% of the collection. The mean score of severity in this young experimental plot was 0.01 ± 0.09 and none of the accessions exceeded a mean score above 0.5 (Table S1).

According to the results in the 14-year-old orchard and the information previously reported by Dapena [26] in a similar study, seven groups of susceptibility to European canker were determined (Table 1). Around 17% of the accessions had a low susceptibility (groups 1 and 2) whereas 29% of the collection proved susceptible to the disease (groups 6 and 7). The remaining genotypes can be considered as little to moderately susceptible cultivars (groups 3, 4, and 5).

### 2.2. Isolates Identification and Pathogenicity Tests

Three different isolates from Asturian apple orchards located 11 km apart were identified by beta tubulin gene sequence [35]. Isolates were named with the GenBank accession number of the trees in which the cankered wood samples were collected (Table 2). In the first pathogenicity test using detached shoots, the ANOVA analysis did not reveal differences in the virulence among isolates regarding the AUDPC (F_2,422_ = 0.51; *p* = 0.49) and infection frequency (F_2,135_ = 3.99; *p* = 0.06). In the second experiment, the pathogenicity was determined by the lesions induced on one-year-old ‘M9’ potted plants. In this trial, the percentage of infected wounds did not vary significantly among isolates (F_2,30_ = 0.88; *p* = 0.52) whereas the isolates OPC304 and BGV344 resulted in statistically different levels of lesion length compared with the isolate P112, which was shown to be the least aggressive (Table 2; F_2,91_ = 2,39; *p* = 0.04). Although no statistical differences were found between isolates OPC304 and BGV344, the isolate OPC304 was chosen for the cultivar resistance evaluation experiments.

### 2.3. Evaluation of Cultivar Resistance on Detached Shoots

**Figure 1:**
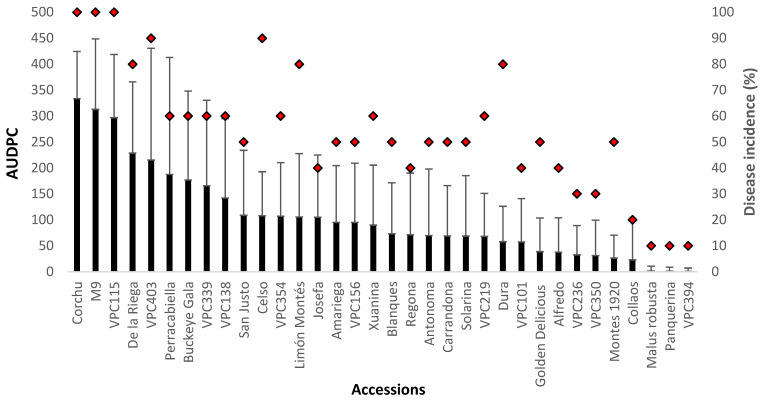
Mean area under disease progress curve (AUDPC; mean ± SD) and disease incidence (proportion of infected wounds as a percentage of the number of inoculated wounds) for detached shoots inoculated with a single *N. ditissima* isolate calculated 42 days post-inoculation. The solid black bars represent AUDPC values whereas the red rhombuses indicate the disease incidence percentage.

In this assay, we found considerable variation in the level of susceptibility among cultivars measured as AUDPC (F_33,311_ = 6.54; *p* < 0.001) and disease incidence (F_33,145_ = 1.95; *p* = 0.004; Figure 1). The accession ‘Corchu’ showed the highest AUDPC value (321.8) whereas the minimum value was recorded in ‘VPC394’ (1.45). The ANOVA test showed that ‘Panquerina’, ‘Collaos’, ‘Montes 1920’, ‘VPC394’, ‘VPC350’, and *Malus robusta* were statistically the most resistant cultivars to *N. ditissima*. We did not find significant differences between blocks or the position of the inoculation scars in the shoot. The correlation between disease incidence and lesion growth (AUDPC) measured by a Pearson’s correlation was strong (r = 0.69), confirming that there is a strong positive correlation between the two parameters.

### 2.4. Evaluation of Cultivar Resistance on Potted Trees

**Figure 2:**
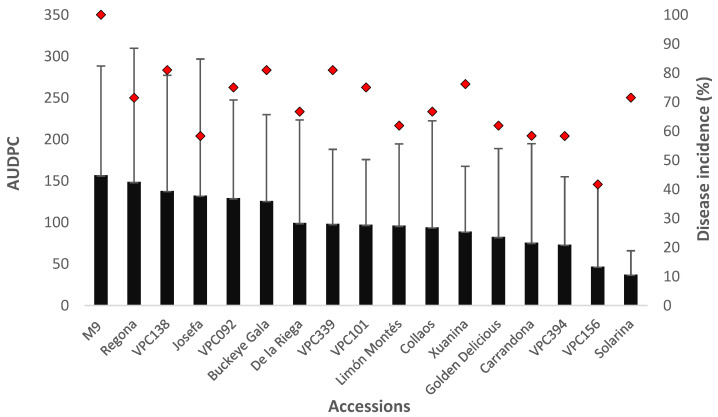
Mean area under disease progress curve (AUDPC; mean ± SD) and disease incidence (proportion of infected wounds as a percentage of the number of inoculated wounds) for 1-year-old potted trees inoculated with a single *N. ditissima* isolate calculated 71 days post-infection. The solid black bars represent AUDPC values whereas the red rhombuses indicate the disease incidence percentage.

In the first trial using inoculum of *N. ditissima* obtained from a single ascospore culture, the GLM analyses indicate that the plant genotype had a significant effect in the length of the lesions and the disease incidence (Table 3). The local cultivar ‘Solarina’ had the highest level of resistance in this test (Figure 2), followed by the accession ‘VPC156’. The rootstock ‘M9’ showed the highest susceptibility in both AUDPC and infection frequency results. Pearson correlation coefficient (r) showed a strong positive relationship between lesion length measured as AUDPC and percentage of infected buds (r = 0.62).

**Figure 3:**
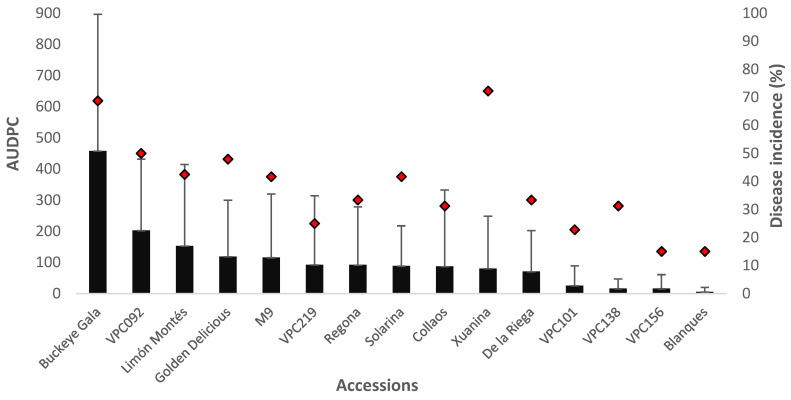
Mean area under disease progress curve (AUDPC; mean ± SD) and disease incidence (proportion of infected wounds as a percentage of the number of inoculated wounds) for 1-year-old potted trees inoculated with field-collected inoculum calculated 164 days post-infection. The solid black bars represent AUDPC values whereas the red rhombuses indicate the disease incidence percentage.

In the second controlled inoculation experiment, for which field-collected conidia was used, the GLM analyses revealed different levels of susceptibility among cultivars by measuring the lesion length (Table 3). However, the differences among cultivars were not statistically significant when the disease incidence values were chosen. ‘Buckeye Gala’ potted trees showed the highest AUDPC and disease incidence values whereas the lowest lesion length was recorded in ‘Blanques’. The rootstock ‘M9’ showed a slightly lower AUDPC values than the known moderately resistant variety ‘Golden Delicious’ (Figure 3). A moderate correlation between lesion length and percentage of infected wounds was observed in this experimental approach (r = 0.48).

### 2.5. Evaluation of Cultivar Resistance Determined by Inoculating Adult Trees under Field Conditions

**Figure 4:**
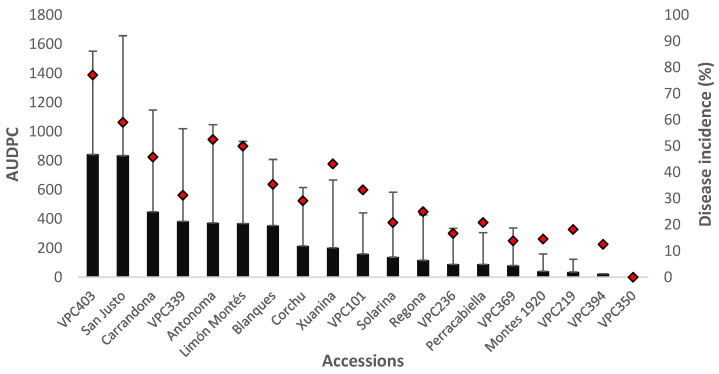
Mean area under disease progress curve (AUDPC; mean ± SD) and disease incidence (proportion infected wounds as a percentage of the number of inoculated wounds) for adult trees inoculated with field-collected inoculum calculated 234 days post-inoculation. The solid black bars represent AUDPC values, whereas the red rhombuses indicate the disease incidence percentage.

According to the data from the field inoculation trial, significant differences in lesion length and disease incidence were observed among 18 accessions (Table 3). The highest number of infected wounds and the most severe lesions were recorded in the accessions ‘VPC403’ and ‘San Justo’ (Figure 4). The accession ‘VPC350’ did not show symptoms of the disease in 48 inoculation points. Other accessions showing a good level of resistance were ‘VPC394’, ‘VPC219’, and ‘Montes 1920’. Although the cultivar ‘Xuanina’ had a high average infection percentage (43%), the development of the lesions was smaller than in other cultivars that had a lower average percentage of infected wounds. The highest correlation between the two components of resistance evaluated in the current work was found in this test (r = 0.83). This assay was generally able to differentiate the most susceptible and the most resistant cultivars, although some inconsistencies were observed. As expected, the susceptible accessions ‘VPC403’ and ‘San Justo’ showed the highest lesion length values, although the field susceptible accession ‘VPC219’ exhibited both low lesion length and disease infection values.

### 2.6. Comparison between Phenotyping Methods for Evaluating Partial Resistance to European Canker

The results from different artificial inoculation methods and types of plant material differed from observations under field conditions in naturally infected trees for the same genotype. None of the controlled treatments resulted in ranks that were similar to the field planted adult trees assay. Therefore, our results suggest a limited relationship between approaches for both resistance parameters (Table S2). The highest correlation for lesion length was found between the trees inoculated directly in the field and the detached shoots experiment (Spearman’s correlation coefficient = 0.69). Spearman correlation between detached shoots and young grafted plants also indicated a statistically significant moderate correlation for both parameters. When comparing the ranking of susceptibility of the different accession using lesion length values, the Spearman’s rank correlation analysis revealed no significant correlations between the field evaluation scores in adult trees and any of the four rapid screening tests (Table 4). As with the disease incidence, a statistically significant Spearman correlation was found between trees inoculated under field conditions and the same adult trees evaluated after several years exposed to natural infections (*p* = 0.02). When we only analysed the five accessions that were tested in all trials, we also observed a lack of statistically significant correlations between the different rapid tests and the information recorded after visual observations on mature field trees (Table S3). In this way, these findings are consistent with those obtained for establishing a ranking of accessions using a larger set of accessions.

## 3. Discussion

### 3.1. Pathogenicity of Local N. ditissima Isolates

The results of the pathogenicity of three locally sampled isolates via controlled inoculations under biotron conditions demonstrated that both the choice of the procedure and plant material have a significant effect on the results. In the detached shoot experiment, the percentage of inoculated buds infected with *N. ditissima* was 29% whereas using potted plants of a single susceptible genotype the percentage reached 67%. The reasons for the lack of evident differences among isolates in the detached shoots experiment are unknown, but were most likely related to the experimental method. The results indicate that the artificial inoculation of detached shoots might not be a suitable alternative to determine the pathogenicity of *N. ditissima* isolates, as there are many factors that can influence the results. The low number of infected buds in this experiment partly hampers the ability to draw further conclusions regarding a possible cultivar-isolate interaction. However, our data seem to be in line with a previous studies reporting that *N. ditissima* isolates seem to not be specific to host cultivars [19,36,37].

Furthermore, the three isolates behaved differently in their pathogenicity when only plants from the canker-susceptible rootstocks ‘M9’ were infected. This result might indicate that the use of a susceptible plant genotype alone may be the preferred procedure to assess the pathogenicity of *N. ditissima* isolates. Additionally, Campos et al. [38] found no evidence of a relationship between the virulence of *N. ditissima* isolates and their geographical origin in Brazil. Further investigations using a larger number of isolates collected in different Spanish regions might help to gain a deeper understanding on the regional specificity among isolates.

### 3.2. Sources for Relative Resistance against N. ditissima among an Extensive Collection of Local Apple Accessions

Under very favourable environmental conditions for the development of *N. ditissima* infections, a significant number of local accessions showed a high level of resistance to the disease. In the process of releasing new cultivars with a high degree of adaptation to the local mild and humid climate, the identification of phenotypic differences among a large pool of genetic resources is a crucial step. To achieve this goal, the conservation of a wide collection of landraces seems particularly relevant for the development of new cultivars [39]. Other countries, as in the case of the United Kingdom, rely their apple production on a small number of cultivars derived from canker-susceptible progenitors [35]. Our results reveal that the SERIDA breeding program has a wide array of suitable material for developing cultivars with an optimal level of resistance to European canker. Additionally, some of the 22 cultivars rated as very resistant in this study also exhibit a high resistance to other diseases and pests as well as good agronomic and technological characteristics [26,40]. Some of these varieties are ‘De la Riega’, ‘Xuanina’, and ‘Solarina’, which were selected by the SERIDA Fruit Research Group and they belong to the Protected Designation of Origin “Sidra de Asturias” since 2002. According to the disease incidence scale developed in this study, we consider that cultivars comprised in groups 1, 2, 3, and 4, which represents 48% of the collection evaluated in this research, are sufficiently resistant for growing cider apples in north-western Spain. It is also important to note that this collection accounts approximately 70% of the local accessions maintained by SERIDA. In this respect, the SERIDA germplasm collection represents a world-class repository, and the breeding program already has access to a broad local genetic diversity without the need to import plant material from other countries to breed European canker resistant cultivars.

Similarly, some of the studied cultivars were evaluated phenotypically by Dapena [26] using the same methodology in other experimental orchards in the same growing area during the winters of 1989/1990 and 1990/1991. These cultivars exhibited equal or lower levels of damage than the ones observed in the current study. These findings lead us to the assumption that cultivars categorised as resistant or moderately resistant in this work are expected to behave similarly regarding their susceptibility to European canker in other commercial orchards in the mild humid climate of the north-western Spain.

Weber and Børve [10] reported regional differences in the perception of canker susceptibility for a given cultivar depending on local factors such as soil or climate. From our perspective, the limitations reported in the literature regarding the suitability of this methodology are rather small in our 14-year-old experimental orchard. Weather conditions, especially rainfall and temperature during infection periods, pressure of inoculum, and orchard management are ideal for the development of the disease. On the basis of this information, we hypothesized that a good correspondence in the level of susceptibility between field resistance scores in 14-year-old trees and any of the rapid screening tests evaluated in this work can provide the basis for implementing innovative and reliable screening tests in apple breeding programs.

### 3.3. Reliability of Phenotyping Methods and Resistance Parameters

All controlled inoculation tests showed statistical differences between cultivars for the two resistance parameters with the only exception being the disease incidence in the potted trees experiment (plants inoculated with field-collected inoculum). This information slightly contrasts with the results reported in a study by Wenneker et al. [41], which indicated that it is easier to find significant differences between cultivars by choosing the disease incidence as the parameter. Scheper et al. [34] and Bus et al. [5] found that lesion length was not a reliable parameter to rank the resistance to European canker among accessions. On the contrary, Ghasemkhani et al. [28] suggested that the infection percentage was less informative than AUDPC under their experimental conditions. Garkava-Gustavsson et al. [42] stated that choosing only the infection percentage may not be sufficient for testing different levels of resistance to European canker. For our set of experiments, the artificial inoculations produced lesions in approximately 48% of the wounds, ranging from 62 to 32%, depending on the test. These percentages are lower than previous studies by Ghasemkhani et al. [28] who reported an average infection percentage across trials and experimental seasons of around 75% and Garkava-Gustavsson et al. [42] who reported a large infection percentage of approximately 95% of the inoculations. However, these latter authors inoculated several susceptible apple varieties commonly used in the dessert apple industry, and therefore higher infection percentages could be expected.

On the other hand, in all but one of the experiments the highest infection frequency coincided with the largest lesion length value. While it is true that moderate-high phenotypic correlations between AUDPC and infection percentage were found in all tests (Pearson´s coefficient varying from 83 to 48%), this parameter does not seem to correlate well for every cultivar. Particularly remarkable are the observations for cv. ‘Xuanina’, as this cultivar showed high infection percentages in all of the controlled experiments, although its AUDPC values were lower than other cultivars ranked below this cultivar regarding the disease incidence results. This cultivar might have developed a resistance mechanism to control the damage of this pathogenic fungi by slowing or halting the development of the lesions (i.e., developing callus around the canker lesion). In this regard, and taking into account that literature often shows contradictory results regarding the effectiveness of the two common resistance parameters, we suggest that both parameters are informative and ideally should be evaluated together in the process of optimizing reliable screening tests. Furthermore, this variable does not require additional experimental work since it can be analysed from the lesion length datasets.

While screening tests can be a valuable tool to establish levels of susceptibility to *N. ditissima*, the reliability of these methods seem not to have a direct relevance to the orchard situation under the humid environmental conditions of north-western Spain. None of the methods resulted in a high correlation rank when the outputs of these tests were compared to the field observations rank. Surprisingly, the highest correlation was found between detached shoots and adult trees inoculated with field-collected inoculum. Since the shoots tested in the biotron were sampled from the same trees, this information might suggest that floral foam might partially reproduce the conditions of living fruit trees during the rather short lifespan of the shoots. Beyond this somewhat unexpected result, the highest correlation with trees previously field phenotyped for *N. ditissima* was found in adult trees inoculated with field-collected inoculum, especially when the disease incidence was chosen as a parameter for resistance.

It is also important to note that results for a given cultivar using similar experimental approaches are often not consistent between studies. For the detached shoots experiment, our results on AUDPC values are consistent with previous studies reporting the relative resistance of *Malus robusta* [19,27,34] and ‘Golden Delicious’ [19,25,28,34,42]. The results also coincide with the susceptibility of ‘M9’ and ‘Gala’ [19,34]. However, several field-resistant cultivars such as ‘De la Riega’ or ‘VPC339’ were classified as susceptible and cultivars categorised as susceptible in the experimental orchard (e.g., ‘Dura’) were scored as moderately resistant in this test. The fact that the resistance ranking of the four genotypes included as international references in this test is in line with previous reports might suggest that a larger collection of accessions must be evaluated in order to confirm the accuracy of this type of procedure.

Regarding the results of potted tree trials, our results are consistent with previously published studies when the inoculum applied to the trees was obtained from a single ascospore culture [19,27]. However, the results regarding the susceptibility of ‘Golden Delicious’ and ‘M9’ plants inoculated with field-collected inoculum were unexpected, since the variety ‘Golden Delicious’ was as susceptible as ‘M9’. These results are somewhat surprising, since all cultivars for both experiments were grafted on the same rootstock and the shoots for the dessert apple varieties were obtained from the same nursery. It is important to highlight that in this experiment, the popular cider apple cultivars ‘De la Riega’, ‘Xuanina’, ‘Collaos’, ‘Solarina’, and ‘Regona’ showed similar lesion length values, which coincide with their also very similar low susceptibility scores in mature trees.

It is important to outline some considerations regarding the evaluation methodology. First, the number of buds inoculated in all controlled experiments seems adequate since lesions did not merge in any of the experimental units (shoots or trees) and we did not identify an effect of the position of the inoculation point in any of the experiments (data not shown). Moreover, canker symptoms were not observed in water-inoculated control replicates, thus latent or naturally occurring infections did not appear during the course of the experiments. However, while we attempted to use the same inoculum concentration, the inoculum dose slightly differed between the experiments, which could have led to inconsistencies in some of the results.

In the absence of fungicide sprays, our field phenotyping results highlight the marked differences in the levels of susceptibility/resistance to *N. dittisima* that apple trees can exhibit depending on their state of development. In fact, only 3 of the 15 accessions that showed symptoms in the juvenile state (4-year-old trees) were classified as highly susceptible in the mature trees assay. This information might suggest that only two growing seasons after planting might not be enough time for *N. ditissima* inoculum to build up evenly across the orchard. These young trees also experienced less wounding as they barely bore fruit and the intensity of pruning was light, thus the entry points to develop European canker infections were scarce. With this in mind and in order to ensure a correct selection of accessions, we advise to conduct visual observation on mature trees exposed to natural infections as the SERIDA Fruit Research Group has done for the past three decades. In short, phenotyping for European canker in mature trees exposed to natural infections under field conditions is a lengthy and time-consuming process. However, speedy screening methods appear to have a limited reproducibility. All assays need to be treated with caution as field screening scores seem not to reliably translate into controlled inoculation experiments. This finding is in accordance with Scheper et al. [34] and Karlström et al. [43] who stated that rapid phenotyping methods are not able to replace experiments in field planted trees. While there is broad consensus on the limited applicability of using detached shoots or potted trees for rapid selection in breeding programmes [19,27,34], the current study found that leaf scar inoculations on the same adult trees (14-year-old) where the natural infections assessment had been conducted can also lead to insufficiently reliable results. Being aware that waiting more than 10 years constrains the development of new releases, further research should focus on optimising the artificial inoculations of younger trees (4-year-old) in the orchard situation. Accordingly, controlled inoculation experiments need further optimization to support breeding programs in selecting cultivars with adequate resistant to European canker.

## 4. Materials and Methods

### 4.1. Field Phenotyping in Young and Mature Apple Trees

#### 4.1.1. Plant Material and Experimental Conditions

A field evaluation of the susceptibility of apple accessions to European canker was conducted in two SERIDA experimental orchards in Asturias (north-western Spain). Trees were trained in a central leader system and managed under organic management practices. No fungicide treatments were applied during the entire lifespan of the orchards in order to allow the development of European canker. Soil and climate conditions in the area were also particularly favorable for the development of the disease.

The first evaluation was carried out in a plot located in Oles (43°31′ N, 5°27′ W) during the winter of 2018/2019. The orchard consisted of 14-year-old trees grafted onto ‘M7’ rootstock (Figure S1) with a planting distance of 5.5 m × 2.5 m. A total of 400 Asturian accessions were assessed with every genotype represented by a single block of two trees. The trees showed a great vigor, a high number of branches and shoots, and they were barely pruned. These conditions point to the existence of an accumulative effect and high disease pressure with abundant inoculum available for infections. Other microclimatic conditions also favored a humid environment in the orchard (e.g., orchard orientation and dense canopies). A completely randomized planting design, with cultivars of different susceptibility levels arranged nearby could also have favored infections between cultivars by aerial rain splash from tree to tree [44]. Furthermore, the orchard is located only 2 km away from the seaside. Ascospores of the fungi have been reported to have a considerable importance in maritime areas as they can be transported hundreds of meters by breezes or air currents [45]. In short, the possibilities to assess the level of resistance/susceptibility in this orchard were optimal.

A disease assessment in naturally infected trees was also conducted in juvenile trees planted in Villaviciosa (43°28′ N, 5°26′ W). This orchard is a duplicate collection of the one in Oles, therefore the same accessions are represented. Two-year-old trees grafted onto ‘M7’ rootstock were planted in winter 2020 at a spacing of 5.5 m × 2.5 m (Figure S2). The visual observation assessment was carried out in blocks containing two trees for each accession. The evaluation was carried out in January 2022, thus the trees were nearly 4-year-old at the time of evaluation. 

#### 4.1.2. Phenotypic Susceptibility Assessment after Natural Infections

To determine the damage caused by *N. ditissima* in the studied trees, visual observations of symptoms in naturally infected trees were carried out using a disease severity scale with six levels (adapted from Lateur and Populer [31] and Dapena [26]). Owing to the wide variety of symptoms observed in mature trees, intermediate values (0.5) were incorporated to ensure that the whole range of symptoms was represented in the evaluation. The level of susceptibility was recorded according to the following an ordinal scale: (i) 0—no visible symptoms; (ii) 1—some cankers but hard to find (low incidence); (iii) 2—visible injuries but its presence is still relatively limited (medium–low incidence); (iv) 3—cankers spread throughout the tree (high incidence); (v) 4—severe damage with a significant number of cankers widespread over the tree (very high incidence and intense lesions); (vi) 5—maximum susceptibility (very high incidence with severe girdling which can result in the death of the tree). 

### 4.2. Isolates Identification and Pathogenicity Tests

#### 4.2.1. Isolates Identification and Inoculum Preparation

Naturally occurring cankers on apple branches infected with *N. ditissima* were collected in three experimental orchards situated in an important apple growing area named as “Comarca de la Sidra” (Asturias, north-western Spain) in December 2017 (Table 2). The orchards are organically managed and, although the radius between the three locations is only 11 km, the characteristic orography of the region provides slightly different microclimate conditions between them.

Small transverse sections from the edges of the infection were cut using either a hacksaw or a Stanley blade. The protocol for the surface sterilization was adapted from Petrini [46] using 70% ethanol and 5% NaOCl solutions. After the sterilization, sections were dried using sterile paper tissue and aseptically dissected using a scalpel. Smaller pieces from the edges of the infection and from the xylem necrotic areas were placed onto PDA plates (39 g potato dextrose agar in 1 litre of distilled water) supplemented with the antibiotic rifamycin and iprodione, both at 20 ppm [47]. Clean cultures were sub-cultured onto SNAY media (1 g potassium dihydrogen phosphate, 1 g potassium nitrate, 0.5 g magnesium sulfate, 0.5 g potassium chloride, 0.2 g glucose, 0.2 g sucrose, 1 g yeast extract, 20 g agar made up to 1 litre with distilled water) and incubated at room temperature for 15–30 days. Spore suspension was prepared by flooding the plate with sterile water. A volume of 50 μL was spread onto water agar plates. A single spore was collected from water agar plates and inoculated onto SNAY plates. Mycelium from single spore cultures was scraped using a sterile toothpick and inoculated in flasks with 20 mL of YPD media (20 g Bacto peptone, 10 g yeast extract, 950 mL of water, 50 mL of 40% w/v glucose). Flasks were incubated in a shaker at a constant 20 °C at 120 rpm for a week. Mycelium was filtered and freeze-dried. A subsample of 30 mg of freeze-dried material was disrupted using metal ball bearings and a tissue lyser for 2 min at 15 Hz. DNA extractions were performed using the Macherey-Nagel NucleoSpin Plant II kit as described in the manufacturer’s handbook. A primer set of a region of the β-tubulin gene of *N. ditissima* was used for the identification of these isolates. Primers and PCR conditions were performed as described in Ghasemkhani et al. [28]. 

#### 4.2.2. Pathogenicity and Virulence of Local Isolates

The pathogenicity of three isolates of *N. ditissima* was tested in two inoculation experiments. In the first experiment, their pathogenicity was investigated using dormant detached shoots from eight cultivars. We chose six local cultivars widely planted in the region: ‘De la Riega’, ‘Solarina’, ‘Limón Montes’, ‘Regona’, ‘Xuanina’, and ‘Collaos’. Additionally, ‘Golden Delicious’ and ‘Buckeye Gala’ (‘Buckeye^cov^Gala’ Simons) were included as international references for comparison. Dormant one-year-old shoots were collected in March 2018, wrapped in moist paper and kept at 4 °C in darkness for 6 weeks. Shoots from the local cultivars were sampled from a local orchard whereas shoots from ‘Buckeye Gala’ and ‘Golden Delicious’ were obtained from a certified nursery (Dalival, France). The mean shoot length was 53.8 ± 8.4 cm. Four days before inoculation, shoots were placed into a biotron in a 16/8 h light/dark cycle where the temperature was not manually controlled (Figure S3). Shoots were pinned in Oasis floral foam placed into a tray containing water with Crystal Clear^®^ Flower Food Clear 300 1% (adapted from Gomez-Cortecero et al. [19]) The base of the shoots was cut by approximately 1 cm once a week. Six shoots from each cultivar were selected and three axillary buds on each shoot were inoculated. A spore suspension from each *N. dittisima* isolate was applied to the wound made in buds placed in the 4th, 9th, and 14th position from the apex. Therefore, six replicates (shoots) with three inoculation points per shoot were used for each individual isolate. The wound was prepared by cutting off the bud with a scalpel and the spore suspension was applied to the wound within 2 min after making the wound and covered with white petroleum jelly (Vaseline^®^), which was removed after 4 days with tissue paper. The inoculum volume for this experiment was 3 µL (with a concentration of 1 × 10^5^ conidia/mL).

In the second experiment, 11 potted plants (3 litres pots) of the rootstock ‘M9’ (M9 Pajam^®^ 2 (Cepiland)) were inoculated with inoculum from each local isolate in August 2018. On each plant, three axillary buds spaced approximately the same distance apart were inoculated following the same methodology described above. An inoculum volume of 5 µL (with a concentration of 7.5 × 10^5^ conidia/mL) of spore suspension was applied to the wounds.

### 4.3. Evaluation of Cultivar Resistance on Detached Shoots

The phenotypic differences in the levels of resistance of a collection of accessions were determined in dormant terminal one-year-old shoots. Shoots were collected from adult trees grown in the same orchard where the natural disease severity assessment had been conducted. The length of the sampled shoots was 43.1 ± 8.3 cm. With the aim of choosing plant material showing different levels of susceptibility, the selection of cultivars was based on their behaviour under field conditions. Thirty-five accessions were evaluated in this study: 31 local cider apple cultivars, 2 dessert apple varieties (‘Buckeye Gala’ and ‘Golden Delicious’), the widely used apple rootstock ‘M9’, and the crab apple *Malus × robusta* (Carrière) Rehder (*M. baccata* × *M. prunifolia*). Shoots from ‘Buckeye Gala’, ‘Golden Delicious’ and ‘M9’ were obtained from a certified nursery (Dalival, France) and shoots from *Malus robusta* were collected from the SERIDA apple repository. Five shoots from each cultivar were inoculated using a single isolate named as OPC304. The experimental conditions and inoculation method were the same as the pathogenicity test using detached shoots, with the only difference being that only two buds were inoculated in this test due to a shorter length of the sampled shoots. Shoots were inoculated by adding 4.5 µL of spore suspension (2.6 × 10^5^ conidia/mL) to the wounds.

### 4.4. Evaluation of Cultivar Resistance on Potted Trees

#### 4.4.1. Inoculation Using In Vitro Conidial Production from a Single Isolate

Dormant one-year-old shoots from a subset of 17 cultivars were grafted onto ‘M9’ rootstock in March 2018. Potted trees (4 litres) were inoculated in mid-October in the same year. The mean growth of the main stem from the graft union was 34.6 ± 8.8 cm. All potted trees were transferred to an unheated biotron 48 h before inoculation (Figure S4). Seven sets of trees in a completely randomised block design were inoculated using a single *N. ditissima* isolate (OPC304). On each plant, three leaves were removed randomly and an inoculum volume of 7.5 µL of spore suspension (4.4 × 10^5^ conidia/mL) was applied to the wounds.

#### 4.4.2. Inoculation Using Field-Collected Inoculum

Fifteen genotypes were chosen to compare the level of susceptibility against European canker after inoculating the plants with field-collected inoculum. In January 2020, one-year-old plants grafted onto ‘M9’ rootstock were inoculated in an unheated biotron. The *N. ditissima* field-produced conidia (henceforth called field inoculum) was prepared by sampling field cankers (Figure S5) four days before the inoculation and were kept at room temperature (18–20 °C). Sporodochia was transferred to distilled water using a binocular dissecting microscope and a sterile scalpel. Four leaves randomly removed were inoculated with an inoculum volume of 7.5 µL of spore suspension (1.05 × 10^5^ conidia/mL).

### 4.5. Evaluation of Cultivar Resistance Determined by Inoculating Adult Trees under Field Conditions

The same trees where the cultivar susceptibility assessment in naturally infected trees had been conducted during the winter of 2017/2018 were inoculated with *N. ditissima* in November 2019. Experimental trees had old lesions of European canker but shoots selected for inoculation did not show observable symptoms of the disease. A suspension (2 × 10^5^ conidia/mL) of field produced conidia was prepared from cankers collected in the same orchard. Four leaf scars, spaced at least 12 cm apart, were made on each shoot by removing leaves. Inoculations were conducted during the natural leaf fall period but due to the different rates of leaf drop among the studied cultivars, bud scars were created using a scalpel. Artificially made leaf scars were inoculated with 20 µL of a *N. ditissima* spore suspension and droplets were spread over the wound to avoid the run-off (adapted from Walter et al. [48]).

### 4.6. Experimental Design and Disease Evaluation Methods

All experiments were arranged in a complete randomized block design. For artificial inoculation experiments, the inoculum was prepared on the day of the inoculation and the spore concentration was adjusted depending on the experiment using a Neubauer haemocytometer. Only macroconidia of *N. ditissima* were counted, as they are were previously assigned to be highly infectious [48]. Conidial germination of fresh inoculum was determined by pipetting 100 μL spore suspension onto glass slides and incubating overnight at (20 °C) and high humidity. The numbers of germinated and non-germinated spores were counted using a Nikon Eclipse 50i compound microscope at 100× magnification. Conidial germination rate from field-collected inoculum ranged between 76 and 94% for different inoculation days.

Supplementary lighting of 10/14 h light/dark (photoperiod of November in the study region) was provided during the course of the experiments in an unheated biotron. For a successful infection, relative humidity in a controlled environment was increased to nearly 100% using overhead misting lines during the first 4 days after inoculation. Misting lines were activated for 15 s twice a day to ensure a sufficient relative humidity. Air temperature and relative humidity were recorded hourly over the course of the experiments using a data logger (EL-USB-2; Lascar Electronics Ltd., Whiteparish, Wiltshire, UK). The data logger in the experimental orchard was placed inside a small Stevenson’s screen and positioned 1.5 m from the ground. The start and end dates of the experiments and the specific environmental conditions for each of them are shown in Table S4.

As a control, leaf scars in one shoot or plant per cultivar were inoculated with sterile water. In all experiments, two parameters were evaluated to quantify the levels of resistance to European canker after artificial inoculations. The disease incidence, also called infection frequency, was determined as the number of infected wounds at the last assessment by the total number of wounds inoculated. The lesion length was measured on a regular basis with a digital calliper from the onset of symptoms and the area under the disease progress curve (AUDPC) was calculated. The number of days between two successive lesion length measurements was the same within each experiment but varied between experiments depending on when the first symptoms were noticed (Table S4). Early lesion symptoms were basically changes in bark colour, with sunken and swollen areas near the inoculation point. If plants or shoots died before the end of the experiment, data from the last lesion assessment were excluded from the statistical analyses.

### 4.7. Statistical Analysis

All analyses were implemented in the R programming environment (R Development Core and Team, version 4.0.4; [49]). Differences in the pathogenicity among *N. dittisima* isolates and the level of susceptibility to this fungus among a collection of apple accessions were analysed using the ‘agricolae’ package [50]. Two-way ANOVA or Linear Mixed Models with either the area under disease progress curve (AUDPC) or the disease incidence as a variable were performed to examine the different levels of susceptibility among accessions and replicates within the same phenotyping experiment. For the detached shoots experiment and the two trials used to determine the relative pathogenicity of isolates, a two-way ANOVA followed by Tukey’s HSD test was used compare means at a significance level of 5%. For the other rapid screening tests, data was analysed using a Linear Mixed Model, which included accession as the fixed effect and the experimental unit (shoot or tree) as a random effect. To stabilize variance, all data were either log or arcsine transformed to meet the criterion for normality prior to statistical analysis. Spearman’s rank correlation analysis was used to compare the ranking of accessions in terms of their susceptibility to *N. ditissima* between different tests.

## 5. Conclusions

To our knowledge, this is the first study comparing the ranking of susceptibility to European canker for the same genotype on adult trees (14-year-old) and juvenile trees (4-year-old) exposed to naturally occurring infections and several artificial inoculation experiments with detached shoots and potted plants. Under high humidity and regular rainfall conditions and, based on observations in adult trees, none of the 400 local apple accessions proved totally resistant to *N. ditissima.* By contrast, only 3.8% of the collection developed symptoms when the same assessment was conducted in young trees. Wide variation in levels of resistance of adult trees to European canker was observed among the investigated apple accessions and around 28% of the collection showed a high level of resistance to the disease. This information reveals sources of resistance among Asturian cider apple accessions and some of these accessions have already been used as parents for breeding new cultivars both with a high level of resistance to European canker and for its own cultivation.

On the other hand, the phenotyping results in controlled experiments were not consistent with visual observations of symptoms in naturally infected trees and only adult trees inoculated with field-collected inoculum showed a moderate correlation, especially when the disease incidence was chosen as a parameter for resistance. While phenotyping for *N. ditissima* resistance in adult trees is a laborious task, the contradictory results in the ranking of cultivar susceptibility in all rapid screening tests suggest that field planted trees maintained in the same location and exposed to natural infections across multiple years seem the preferred option to determine the relative level of resistance to *N. ditissima* in areas that are environmentally favourable to the disease.

## Figures and Tables

**Table 1:** Levels of field resistance to European canker among 400 Asturian apple accessions after phenotypic observations in 14-year-old apple trees exposed to natural infections in Oles (north-western Spain). The range of scores for disease severity, number of cultivars and percentage of the germplasm collection comprised in each level of field resistance. Means ± standard errors within each group are also shown.

Susceptibility Group	Field Resistance Classification	Range of Scores for Disease Severity	Number of Cultivars	Mean ± Standard Error	Percentage of the Collection
1	Very resistant	0–0.5	22	0.34 ± 0.04	5.5
2	Resistant	0.51–1	43	0.88 ± 0.02	10.75
3	Moderately resistant	1.01–1.5	51	1.43 ± 0.02	12.75
4	Intermediate	1.51–2	75	1.88 ± 0.01	18.75
5	Moderately susceptible	2.01–2.75	94	2.5 ± 0.02	23.5
6	Susceptible	2.76–3.25	82	3.08 ± 0.01	20.5
7	Highly susceptible	3.26–5	33	3.68 ± 0.03	8.25

**Table 2:** Isolate name, origin (location and year of collection), and host of the *N. ditissima* isolates used to determine their pathogenicity on a series of controlled inoculations experiments on ‘M9’ potted plants. The disease incidence (expressed as the percentage of infected wounds) and AUDPC (area under the disease progress curve) values were obtained 42 days after the inoculation. Values followed by a different letter differ significantly (*p* < 0.05) according to the ANOVA analysis.

Isolate Name	Location	Year of Collection	Host	Disease Incidence (%)	AUDPC (mm)
OPC304	Oles	2017	Local accession	66.7 a	201.83 a
P112	Priesca	2017	‘Carla’	60.6 a	59.25 b
BGV344	Villaviciosa	2018	‘Urtebi’	72.7 a	136.17 ab

**Table 3:** Results of generalized linear models (GLM) analyses for AUDPC and disease incidence (%) in three artificial inoculation tests. Degrees of freedom (Df) stands for the number of accessions evaluated in each experiment. The letter “n “stands for the number of inoculation points for the AUPDC analysis and the number of inoculated shoots (adult trees test) or potted trees in the disease incidence (%) analysis.

Test	Variable	Df	n	*p*
Potted trees (single isolate inoculum)	AUDPC	16	300	<0.001
	Disease incidence (%)	16	108	0.004
Potted trees (field-collected inoculum)	AUDPC	15	300	0.011
	Disease incidence (%)	15	140	0.05
Adult trees (field-collected inoculum)	AUDPC	18	850	<0.001
	Disease incidence (%)	18	221	<0.001

**Table 4:** Spearman´s correlation coefficients between different European canker phenotyping tests using AUDPC or disease incidence (in brackets) as phenotyping variables in a collection of apple accessions in Villaviciosa (north-western Spain). NI, SI, and FI stand for natural infections, single isolate, and field inoculum, respectively. Bold font indicates that the correlation is significant at *p* < 0.05.

	Field Assessment (NI)	Detached Shoots (SI)	Potted Trees (SI)	Potted Trees (FI)	Adult Trees (FI)
Field assessment (NI)					
Detached shoots (SI)	0.23 (0.26)				
Potted trees (SI)	0.15 (0.06)	**0.57 (0.50)**			
Potted trees (FI)	0.13 (−0.38)	0.06 (0.41)	0.23 (0.24)		
Adult trees (FI)	0.41 **(0.51)**	**0.69 (0.49)**	0.14 (0.33)	−0.21 (0.36)	

## Data Availability

Data sharing upon request.

## Supplementary Materials

The following supporting information can be downloaded at: https://www.mdpi.com/article/10.3390/plants11091145/s1, Figure S1. Image of a 14-year-old apple phenotyped for resistance to *N. ditissima* in January 2019 in a SERIDA experimental orchard at Oles (north-western Spain); Figure S2. Image of a 4-year-old apple tree phenotyped for resistance to *N. ditissima* in January 2022 in a SERIDA experimental orchard at Villaviciosa (north-western Spain); Figure S3. Image of detached shoots from apple cultivars pinned in Oasis floral foam and placed into a tray containing water. Shoots were inoculated with a spore suspension of *N. dittisima*. The experiment was carried out in an unheated biotron with supplementary lighting of 10/14 h light/dark; Figure S4. Artificially inoculated one-year-old potted trees in replicated experiment conducted in an unheated biotron with supplementary lighting of 10/14 h light/dark. Potted trees were inoculated with a spore suspension of *N. dittisima*; Figure S5. Red perithecia on the surface of old canker lesion in December 2019 in Villaviciosa (north-western Spain); Table S1. Mean level of disease severity after phenotypic observations in young and adult apple trees exposed to natural infections of *N. ditissima* in two nearby experimental orchards in Asturias (north-western Spain). Adult and young trees were respectively 14-year-old and 4-year-old at the time of the evaluation. The level of susceptibility was recorded in 400 accessions according to an ordinal scale with six levels. The table only shows the results of the young trees that exhibited symptoms in the field observation test; Table S2. Mean level of disease severity in naturally infected trees (14-year-old trees) and mean AUDPC (area under the disease progress curve) and disease incidence (%) for a set of apple cultivars inoculated with *N. ditissima* in a series of controlled assays. The number of days post-inoculation for calculating AUDPC values varied depending on the experiment (see captions of Figure 1, Figure 2, Figure 3 and Figure 4 in the main text). NI, SI, and FI stands for natural infections, single isolate, and field inoculum, respectively; Table S3. Spearman´s correlation coefficients between different tests using AUDPC or infection frequency (in brackets) as phenotyping variables in a set of five apple cultivars (‘Limón Montés’, ‘VPC101’, ‘Solarina’, ‘Regona’ and ‘Xuanina’) in Asturias (north-western Spain). Field assessment was conducted in adult trees (14-year-old). NI, SI, and FI stands for natural infections, single isolate, and field inoculum, respectively. Bold font indicates that the correlation is significant at *p* < 0.05; Table S4. The start and end dates of the experiments and environmental conditions during the course of the experiments. Evaluation days refers to the days on which the lesion length in the experimental units was recorded relative to number of days post-inoculation. SI and FI stands for single isolate and field inoculum, respectively.
