# Supplementary material for: Susceptibility of Cider Apple Accessions to European Canker—Comparison between Evaluations in Field Planted Trees and Rapid Screening Tests
Source: Plants (Basel). 2022 Apr 23;11(9):1145. doi: 10.3390/plants11091145 (PMC9103470; doi:10.3390/plants11091145)
Supplement: Supplementary file 1 [file plants-11-01145-s001.zip › plants-1663623-supplementary.pdf]

Supplementary materials for "Susceptibility of Cider Apple to *Neonectria ditissima* – Comparison between Evaluations in Field Planted trees and Rapid Screening Tests"

Álvaro Delgado [1]*, Belén García-Fernández[1], Antonio Gómez-Cortecero[2] and Enrique Dapena[1]

[1] Servicio Regional de Investigación y Desarrollo Agroalimentario (SERIDA), Asturias, Spain
[2] NIAB, Lawrence Weaver Rd, Cambridge, CB3 0LE, United Kingdom
* Correspondence: alvaro.delgadodelgado@serida.org

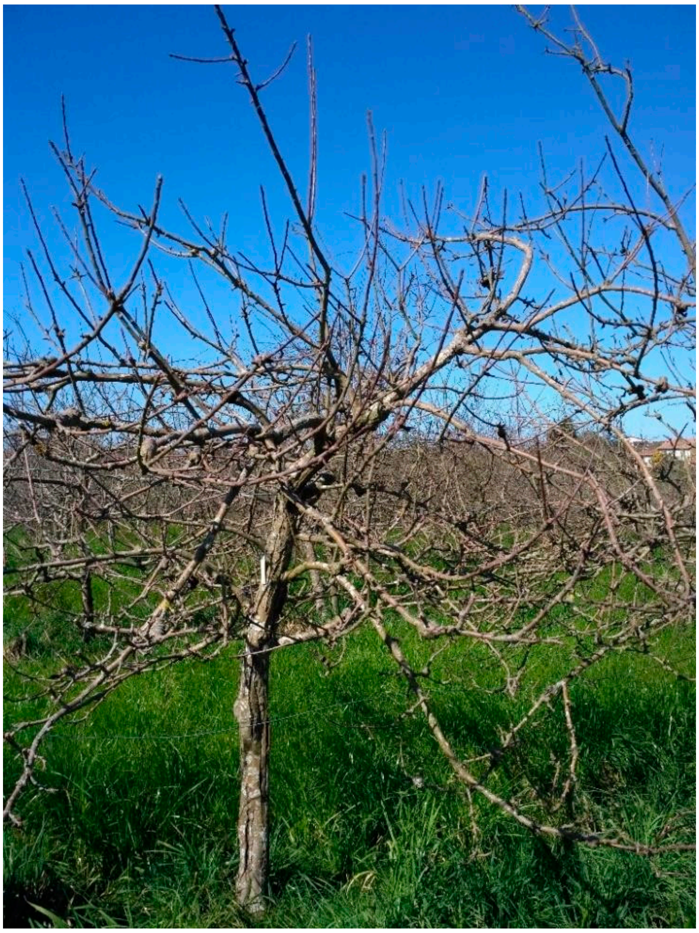

Figure S1. Image of a 14-year-old apple phenotyped for resistance to *N. ditissima* in January 2019 in a SERIDA experimental orchard at Oles (north-western Spain).

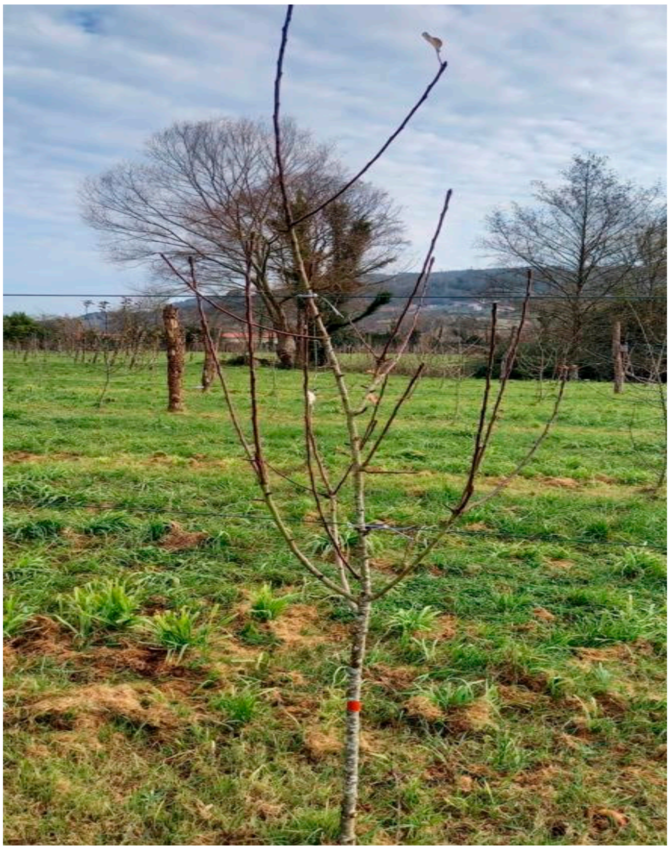

Figure S2. Image of a 4-year-old apple tree phenotyped for resistance to *N. ditissima* in January 2022 in a SERIDA experimental orchard at Villaviciosa (north-western Spain).

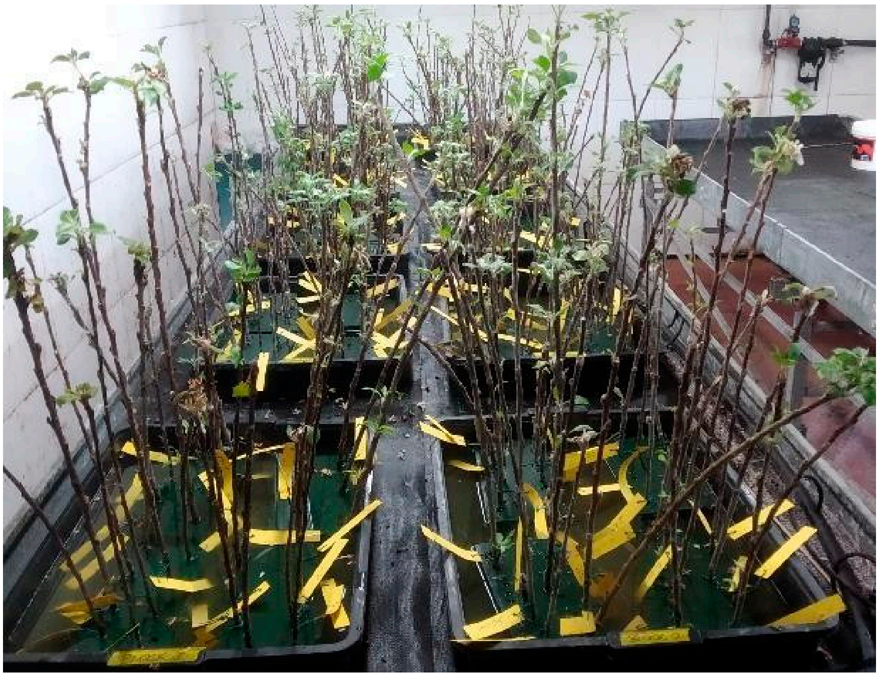

Figure S3. Image of detached shoots from apple cultivars pinned in Oasis floral foam and placed into a tray containing water. Bud scars were inoculated with a spore suspension of *N. dittisima*. The experiment was carried out in an unheated biotron with supplementary lighting of 10/14h light/dark.

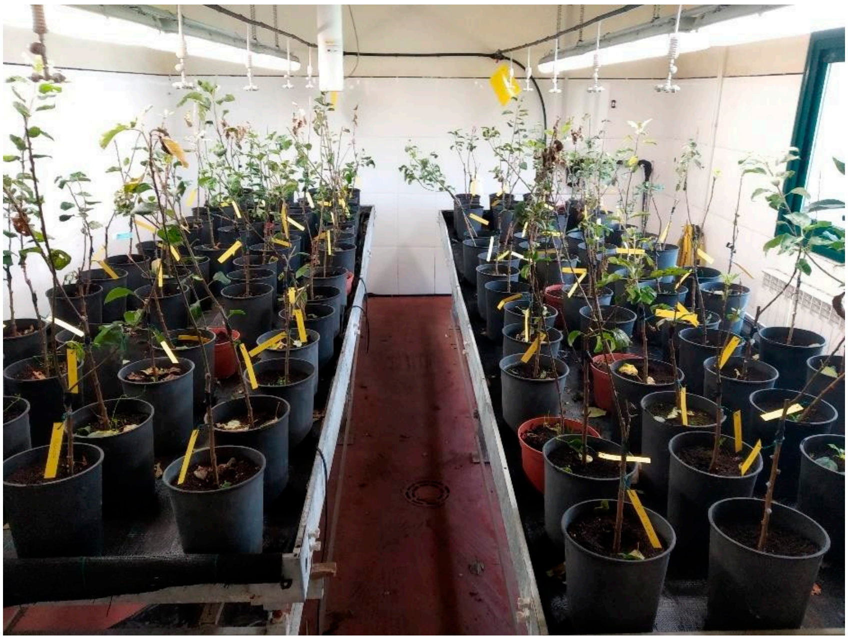

Figure S4. Artificially inoculated one-year-old potted trees in replicated experiment conducted in an unheated biotron with supplementary lighting of 10/14h light/dark. Potted trees were inoculated with a spore suspension of *N. dittisima*.

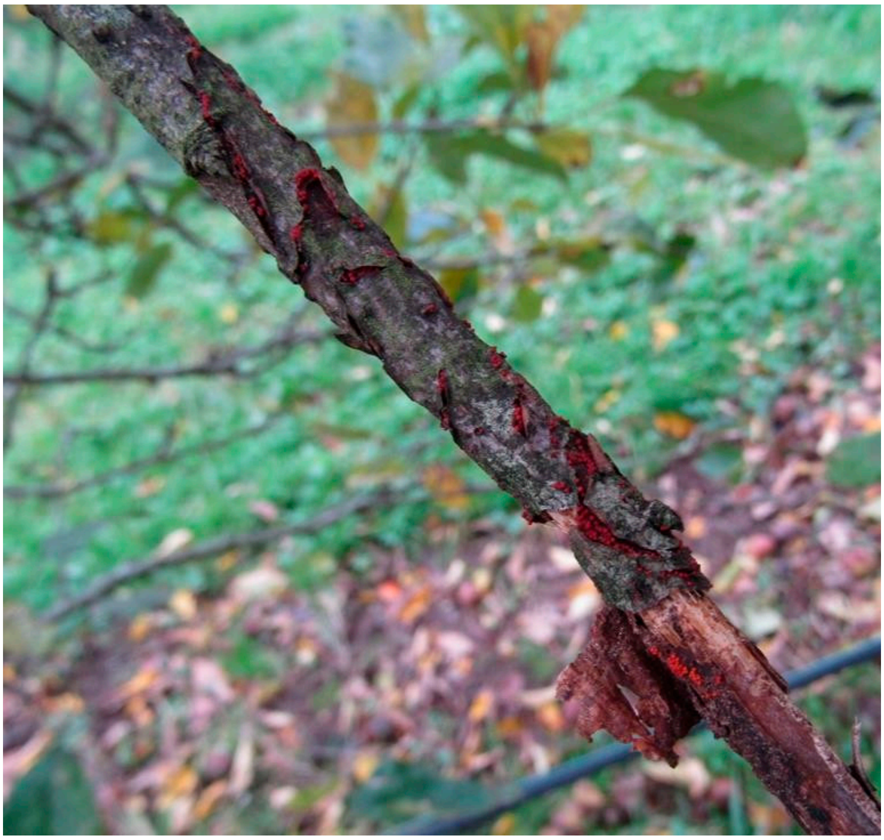

Figure S5. Red perithecia on the surface of old canker lesion in December 2019 in Villaviciosa (north-western Spain).

Table S1. Mean level of disease severity scores after phenotypic observations in young and adult apple trees exposed to natural infections of *N. ditissima* in two nearby experimental orchards in Asturias (north-western Spain). Adult and young trees were respectively 14-year-old and 4-year-old at the time of the evaluation. The level of susceptibility in 400 accessions was recorded according to an ordinal scale with six levels. The table only shows the results of the young trees that exhibited symptoms in the field observation test.

**Cultivar**	**Mean disease severity scores**	
	**14 – year – old**	**4 – year – old**
Antonoma	3.25	0.25
Corchu	1.75	0.25
Dulcina	2.75	0.50
VPC005	1.50	0.25
VPC026	3.00	0.50
VPC073	0.25	0.50
VPC077	1.00	0.25
VPC085	0.50	0.25
VPC108	2.75	0.50
VPC139	3.00	0.25
VPC312	3.00	0.25
VPC354	3.75	0.25
VPC355	3.75	0.50
VPC403	3.75	0.25
VPC449	2.25	0.25

Table S2. Mean level of disease severity in naturally infected trees (14-year-old trees) and mean AUDPC (area under the disease progress curve) and disease incidence (%) for a set of apple cultivars inoculated with *N. ditissima* in a series of controlled assays. The number of days post-inoculation for calculating AUDPC values varied depending on the experiment (see captions of Figures 1, 2, 3 and 4 in the main text). NI, SI and FI stands for natural infections, single isolate and field inoculum, respectively.

Cultivar	Field assessment (NI)	Detached Shoots (SI)		Potted trees (SI)		Potted trees (FI)		Adult trees (FI)	
		AUDPC	%	AUDPC	%	AUDPC	%	AUDPC	%
Alfredo	2.75	37.69	40.0						
Amariega	1.75	95.18	50.0						
Antonoma	3.25	68.90	50.0					373.83	52.50
Blanques	0.50	72.51	50.0			5.62	15.00	355.46	35.42
Carrandona	2.75	69.30	50.0	75.57	58.33			450.23	45.83
Celso	3.25	106.14	90.0						
Collaos	2.25	23.75	20.0	94.20	66.67	86.93	31.25		
Corchu	1.75	321.80	100					215.26	29.17
De la Riega	0.50	221.89	80.0	99.38	66.67	70.54	33.33		
Dura	3.25	56.36	80.0						
Gala		171.47	60.0	126.05	80.95	457.72	68.75		
Golden		37.56	50.0	82.59	61.90	118.86	47.92		
Josefa	2.75	103.48	40.0		58.33				
Limón Montés	1.00	103.01	80.0	96.25	61.90	152.96	42.50	369.67	50.00
M9		302.18	100	145.89	100.00	115.87	41.67		
Montes 1920	0.50	25.77	50.0					41.31	14.58
Panquerina	1.25	1.80	10.0						
Perracabiella	1.75	186.62	60.0					88.91	20.83
Regona	0.50	68.04	40.0	148.89	71.43	91.93	33.33	116.76	25.00
San Justo	4.00	108.93	50.0					835.18	59.09
Solarina	0.25	66.57	50.0	37.33	28.57	88.81	41.67	138.26	20.83
VPC092	3.00			129.45	75.00	202.68	50.00		
VPC101	3.00	57.51	40.0	97.34	75.00	20.75	22.73	159.71	33.33
VPC115	1.50	297.68	100						
VPC138	1.75	139.41	60.0	138.10	80.95	16.13	31.25		
VPC156	3.00	91.88	50.0	46.92	41.67	16.05	15.00		
VPC219	4.00	66.61	60.0			92.58	25.00	37.01	18.18
VPC236	0.50	32.15	30.0					89.07	16.67
VPC339	0.50	163.71	60.0	98.46	80.95			384.76	31.25
VPC350	0.50	29.96	30.0					0.00	0.00
VPC354	3.75	105.57	60.0						
VPC369	2.25							80.12	13.89
VPC394	0.25	1.46	10.0	73.37	58.33			24.14	12.50
VPC403	3.75	210.41	90.0					842.70	77.08
Xuanina	0.50	88.53	60.0	89.14	76.19	80.32	72.22	202.95	43.18

Table S3. Spearman´s correlation coefficients between different tests using AUDPC or infection frequency (in brackets) as phenotyping variables in a set of 5 apple cultivars ('Limón Montés', 'VPC101', 'Solarina', 'Regona' and 'Xuanina') in Asturias (northwestern Spain). Field assessment was conducted in adult trees (14-year-old). NI, SI and FI stands for natural infections, single isolate and field inoculum, respectively. Bold font indicates that the correlation is significant at $p < 0.05$.

	**Field assessment (NI)**	**Detached shoots (SI)**	**Potted trees (SI)**	**Potted trees (FI)**	**Adult trees (FI)**
**Field assessment (NI)**					
**Detached shoots (SI)**	-0.39 (-0.28)				
**Potted trees (SI)**	0.14 (0.44)	0.01 (-0.07)			
**Potted trees (FI)**	-0.63 (-0.59)	0.82 (0.48)	0.02 (0.07)		
**Adult trees (FI)**	0.01 (0.15)	**0.88** (0.81)	-0.05 (0.49)	0.70 (0.42)	

Table S4. The start and end dates of the experiments and environmental conditions during the course of the experiments. Evaluation days refers to the days on which the lesion length in the experimental units was recorded relative to number of days post-inoculation. SI and FI stands for single isolate and field inoculum, respectively.

**Experiment**	**Start date**	**End date**	**Temperature (°C)**	**Relative humidity (%)**	**Evaluation days (days after the inoculation)**
Detached shoots (SI)	29-May-18	10-Jul-18	22.91	89.16	21, 28, 35, 42
Potted trees (SI)	11-Sep-18	21-Nov-18	17.45	97.45	50, 57, 64, 71
Adult trees (FI)	27-Nov-19	16-Jul-20	13.17	82.96	174, 204, 234
Potted trees (FI)	13-Jan-20	26-Jun-20	15.66	79.34	120, 142, 164
